# Effect of Fermented Matrix on the Color and Stability of Strawberry and Blueberry Anthocyanins during the Storage of Fruit Yogurts and Soy-Based and Bean-Based Fruit Yogurt Alternatives

**DOI:** 10.3390/molecules28176222

**Published:** 2023-08-24

**Authors:** Iwona Ścibisz, Małgorzata Ziarno

**Affiliations:** 1 Division of Fruit, Vegetable and Cereal Technology, Institute of Food Sciences, Warsaw University of Life Sciences WULS-SGGW, 161 Nowoursynowska Str., 02-787 Warsaw, Poland; 2 Division of Milk Technology, Institute of Food Sciences, Warsaw University of Life Sciences WULS-SGGW, 161 Nowoursynowska Str., 02-787 Warsaw, Poland; malgorzata_ziarno@sggw.edu.pl

**Keywords:** anthocyanins profile analysis, yogurt-type product, plant-based yogurt alternatives, cold storage, natural pigments

## Abstract

The effect of the fermented matrix on the color and the stability of anthocyanins contained in strawberry (*Fragaria ananassa* D.) and highbush blueberry (*Vaccinium corymbosum* L.) preparations for fruit yogurts, as well as soy-based (*Glycine max* L. Merr.) and bean-based (*Phaseolus vulgaris* L.) yogurt alternatives, stored for 8 weeks, was evaluated. To produce the fermented bean matrix, germinated seeds of white and black beans were used. The obtained fermented matrices had similar pH levels, while the soy-based and black bean-based yogurt alternatives were characterized by their high content of isoflavone aglycones and phenolic acids. The degradation of anthocyanins in strawberry and blueberry fermented products during storage followed first-order reaction kinetics. Significant differences were found depending on the fermented plant-based matrix. The fermented soy-based matrix demonstrated the highest T_1/2_ values for total anthocyanins (26.3 and 88.8 weeks for strawberry and blueberry products, respectively), whereas the yogurts exhibited the lowest values (13.3 and 49.3 weeks for strawberry and blueberry products, respectively). In the comparison of anthocyanin degradation during the storage of bean-based products, the pigments in the matrix obtained from fermented black beans showed better stability. During storage, the loss of anthocyanins was higher in strawberry products than in blueberry products, particularly with respect to malvidin and petunidin derivatives and acylated anthocyanins, which exhibited high stability. The total color difference (ΔE*) of blueberry plant-based products after an 8 week storage period ranged from 1.1 to 1.5. This data suggests that the addition of a coloring ingredient for industrial production may not be required.

## 1. Introduction

Color plays a significant role in assessing the quality of fruit products. It is influenced by the natural colorants present in the food as well as compounds that may form during processing and storage. In the case of strawberry- and blueberry-based products, anthocyanins are the primary colorants. These compounds are known to be unstable and prone to degradation during storage [1,2,3]. Several factors affect the stability of anthocyanins, including their chemical structure, concentration, storage temperature, oxygen exposure, and light exposure [4,5]. The composition of the food itself also plays a crucial role, with factors such as pH, water activity, copigment content, sugars, proteins, fats, metal ions, ascorbic acid, and enzymatic activity [6,7,8]. Fruit yogurts pose particular challenges to maintaining color stability during storage, despite typically being refrigerated for relatively short periods [3]. This is primarily due to the high pH of fermented dairy products, the limited amount of anthocyanins in fruit preparations, and the presence of certain yogurt ingredients (such as hydrogen peroxide formed during fermentation), that can accelerate anthocyanin degradation and lead to polymer formation. The stability of anthocyanins during the storage of fruit yogurts is generally low [9,10,11]. To enhance and stabilize the color of these products during storage, producers of fruit yogurts have been adding coloring substances or extracts obtained from plants containing betalains or acylated anthocyanins such as red beetroot, hibiscus, red radish, black carrot [12]. However, due to increasing consumer demand for clean-label products, it is important to determine the factors that can minimize anthocyanin degradation during fruit yogurt storage. This would enable the creation of products with a simple composition while maintaining color stability. Interest in anthocyanin pigments extends beyond their coloring properties, as they also offer potential health benefits to consumers. These benefits include protection against acute liver injury, cardiovascular diseases, and intracellular oxidation [4]

In recent years, there has been a rise in alternative products to dairy-fermented foods made from legumes, such as soybeans and beans [13,14,15]. This trend is driven by an increasing population of people who limit or avoid milk and milk products. Motivations for this shift include concerns about animal welfare, the negative environmental impact of dairy production, and health issues, such as cow’s milk protein allergies and lactose intolerance [12]. Producers often add fruit preparations to improve the flavor of fermented plant-based products. These, similar to dairy yogurts, can cause color changes and pose challenges in maintaining the stability of anthocyanin pigments [4,16]. According to EU food legislation, plant-based yogurt alternatives cannot be referred to with dairy-associated names to avoid misleading consumers. “Yogurt” is exclusively derived from milk, where milk is defined as “the mammary secretion” [17].

Numerous publications have explored the factors influencing anthocyanin stability in various fruit industry products, such as juices, frozen foods, jams, and dried fruits [18]. However, there is limited information available on the factors affecting anthocyanin stability during the storage of fruit yogurts. Studies conducted on yogurts have indicated that the stability of anthocyanin compounds is influenced by factors such as the yogurt’s fat content [19], the presence of proteins and hydrocolloids [1], and the addition of ferulic acid [3]. In our previous research, we demonstrated the effect of specific lactic acid bacteria strains on the stability of anthocyanins during yogurt storage [20]. However, to the best of our knowledge, no published studies have compared the loss of anthocyanins in fruit preparations depending on the fermentation matrix, including milk, soybeans, and white and black beans. Such studies would provide insights into whether the composition of the matrix affects anthocyanin stability, enabling producers to adjust the fruit preparation composition accordingly.

Therefore, the aim of this study was to evaluate the effect of the fermented matrix, specifically yogurts and plant-based yogurt alternatives derived from soybeans, black beans, and white beans, on the stability of anthocyanins in strawberry and blueberry preparations during an 8 week cold storage period. Additionally, the fruit preparations, yogurts, and plant-based yogurt alternatives were characterized to assess the relationship between anthocyanin stability and the components present in the fermented matrix.

## 2. Results and Discussion

### 2.1. Physicochemical Composition of Fruit Preparations, Yogurt, and Plant-Based Yogurt Alternatives

The physicochemical composition of the strawberry and blueberry preparations is presented in Table S1. The soluble solids content in the fruit preparations was slightly higher than assumed in the recipe (38%). This discrepancy may be due to the additional pasteurization of the products after the soluble solid correction of the preparations. This process could potentially lead to a partial inversion of sucrose, resulting in an overestimation of refractometer readings. A similar effect was observed in research conducted by Kumar et al. (2022) [21] where the hydrolysis of sucrose to glucose and fructose increased the soluble solids content in beverages. Additionally, a slightly higher acidity was observed compared to the assumed recipe (0.65 g/100 g). The slight increase in titratable acidity during the production of fruit preparations could be attributed to the formation of acidic compounds resulting from the degradation of ascorbic acid, degradation and/or oxidation of reducing sugars, or the de-esterification of the methoxyl group of pectin to pectic acid [21,22]. The pH of the strawberry preparation produced was 3.52, while that of the blueberry preparation was 3.62. Several factors influenced the observed pH of the fruit preparation, including the amount of added fruit, their degree of maturity, varieties, ripening and storage conditions, and the amount of added citric acid during the production of the preparations.

Regarding the color parameters, the blueberry preparation exhibited lower lightness and yellowness values (L* and b* parameters) and a higher intensity of red color (a* parameter), compared to the strawberry preparation. The color of fruit products depends on the amount and structure of anthocyanins present in the fruits used for production, as well as the pH of the product and changes occurring during thermal processing (degradation of anthocyanins and formation of Maillard reaction products) [23]. The content of sugars and organic acids in the fruit preparations depended not only on the composition of the fruit but also on the ingredients added during production, such as sucrose and citric acid. The content of reducing sugars could also be higher due to partial hydrolysis of the added sucrose. The concentration of sucrose was higher in the strawberry preparations compared to the blueberry preparations, which could be attributed to the fact that sucrose constitutes more than 22% of the total sugars in strawberries, while in blueberries it comprises less than 0.4% [24].

The relatively high content of citric acid in the preparation, with 0.51 g/100 g in the strawberry preparation and 0.68 g/100 g in the blueberry preparation, can be attributed to the significant amounts of citric acid naturally present in these fruits, as well as the use of citric acid during production to regulate the acidity of the preparations. The semifinished products obtained from blueberries contained small amounts of malic acid, consistent with previous publications stating that malic acid in blueberry fruits constitutes less than 4% of total acids [24]. The total content of vitamin C in the strawberry and blueberry preparations was 22.6 mg/100 g and 2.7 mg/100 g, respectively. The observed concentration of vitamin C in the fruit preparations was affected by the natural content of this vitamin in the fruits, as well as the losses that occurred during production. Dehydroascorbic acid accounted for 14% and 44% of the total vitamin C contents in the strawberry and blueberry preparations, respectively. Comparing these results with previous publications on fruit [25,26], where dehydroascorbic acid accounted for 8% of strawberries and 19% of blueberries, it can be concluded that the production process of the preparations likely led to the oxidation of ascorbic acid and an increase in the content of dehydroascorbic acid. The strawberry preparation exhibited a significantly lower content of total phenolics compared to that obtained from the blueberry fruit. A similar content of polyphenols in a strawberry preparation was obtained in research conducted by Oliveira et al. (2015) [1].

Table S2 shows the physicochemical composition of yogurts and soybean, black, and white bean-based yogurt alternatives. The fermentation process led to all products having a pH lower than 4.6. Yogurts exhibited the lowest pH, which aligns with the findings of Madsen et al. (2021) [27] who demonstrated that commercial yogurt has a lower pH than soy-based yogurt alternatives. The rate of acidification depends on the strain’s ability to utilize the different sugars present in the plant-based and milk matrices. Several studies indicate that *S*. *thermophilus* can metabolize glucose, lactose, and fructose, while the utilization of other sugars (sucrose and raffinose) shows variable profiles [28]. When comparing the colorimetric parameters, yogurts had the highest L* parameter and appeared the whitest among the tested products. The soy- and bean-based yogurt alternatives exhibited much lower brightness, potentially due to the distinct light-scattering properties of milk fat globules and milk casein micelles compared to plant proteins [29]. The data indicate that the black bean-based yogurt alternative had the highest value of the a* parameter, followed by the white bean-based yogurt alternative and yogurt. The negative value of the a* parameter suggests that soy-based yogurt alternatives possess a green color feature. Mei et al. (2017) [30] also reported a negative contribution of the a* parameter in soy-based yogurt alternatives. The b* values were positive in all samples, with the white bean-based yogurt alternative having the highest value for this coordinate. All tested samples displayed a yellowish color, which could be a result of Maillard reactions occurring during the heating process [31].

The examined samples exhibited varying amounts of mono- and oligosaccharides. Milk yogurts contained lactose and galactose in the highest amounts, while plant-based yogurt alternatives were dominated by stachyose and sucrose. These results are consistent with those reported by Granito and Álvarez, (2006) [13] for fermented beans (*Phaseolus vulgaris*) and Wang et al. (2003) [14] for fermented soy-based beverages. The content of saccharides in fermented milk and plant-based alternatives depends on their initial content in the raw materials, the changes during processing (the efficiency of extraction), the metabolic capability of the bacterial strains, and the fermentation time. The lactic acid content in the tested fermented products ranged from 0.53 g/100 g for white bean-based yogurt alternatives to 0.60 g/100 g for yogurts. Despite using different plant-based matrices for fermenting the yogurt alternatives, there was no significant difference in lactic acid content. However, all fermented products used a starter inoculum containing *Streptococcus thermophilus* and *Lactobacillus delbrueckii* subsp. *bulgaricus* and the same fermentation time was employed for each product. Granito et al.’s (2003) [32] research showed that fermentation time affects acidity during bean fermentation. Similarly, studies on soy-based beverages have shown significant differences in acidity when fermented with different strains of lactic acid bacteria [14].

The content of ascorbic acid and dehydroascorbic acid in the natural yogurt was much lower than in the fruit preparations, with the highest value observed in the yogurt alternative made from black beans (1.53 mg/100 g). Comparing the vitamin C content of yogurt (0.382 mg/100 g) with that of raw milk (1.95 mg/100 g) [33], it can be assessed that significant changes in vitamin C content occur during yogurt production. However, it should be considered that the sterilization process was used in milk production and vitamin C is a heat-sensitive compound. In addition, lactic acid bacteria used in fermentation can produce hydrogen peroxide (especially *L. delbrueckii* subsp. *bulgaricus*), which can react with vitamin C and cause degradation [34]. The highest content of vitamin C in bean-based yogurt alternatives may be related to the germination process, which increases the content of vitamin C in black beans [35]. Moreover, in the production of plant-based yogurt alternatives, a significant amount of water is added to the homogenized seeds, which also affects the vitamin C content. Yogurt had the highest crude protein and fat content, while the yogurt alternatives made from white beans had the lowest. The content of both protein and fat largely depended on the raw material used to produce the fermented products. In the production of plant-based yogurt alternatives, the content of both protein and fat content was influenced by dilution with water, and in the case of beans, by the germination process. Changes in protein content are related to protease activity during seed germination [35].

Table S3 presents the physicochemical composition of yogurts and soybean, black bean, and white bean-based yogurt alternatives, all of which have been enhanced with the addition of strawberry and blueberry preparations. The pH and total acidity of the fruit yogurts examined did not show a significant difference between the blueberry and strawberry yogurts. The fructose and glucose contents were significantly higher in blueberry yogurts than in strawberry yogurts, which is a difference related to the higher sugar content observed of these sugars in the blueberry preparations. In addition, significantly higher values of glucose were recorded in fruit yogurts, which may be related to the higher glucose content observed in natural yogurts (Table S2). The sucrose content in the tested products varied, ranging from 4.15 g/100 g in blueberry yogurt to 6.37 g/100 g in strawberry soy-based yogurt alternatives. This variation was found to be related to the high sucrose content in strawberry preparations and the soy products used. Lactose was determined only in fruit yogurts, in which raffinose and stachyose were not detected, similar to products that did not contain added fruit. Notably, soy-based yogurt alternatives with both strawberry and blueberry preparations had the highest raffinose and stachyose contents. In terms of acid content, there were no significant differences between the citric and malic acid levels in the analyzed yogurts. Similar relationships were observed in the fruit preparations, where blueberry products contained higher citric acid and lower malic acid content compared to strawberry preparations. Fruit yogurts showed the highest lactic acid content, with an average of 0.425 g of lactic acid per 100 g. Fermented products enriched with blueberry preparations, especially those obtained from soybeans and black beans, were characterized by high phenolic content. In contrast, fruit yogurts exhibited the lowest phenolic content among all the products examined. During the determination of the total phenols content, the nonhydrolyzed casein, which could react with the Folin–Ciocalteu reagent, was removed from the yogurt samples. It is important to note, however, that the measured content of phenolic compounds in yogurt might be overestimated. This is due to the presence of amino acids and whey proteins in the samples, which can also react with the Folin–Ciocalteu reagent.

### 2.2. Content of Isoflavone-Aglycones, Anthocyanins, and Phenolic Acid in Plant-Based Yogurt Alternatives

Table 1 provides the concentration of isoflavone aglycones in plant-based yogurt alternatives. It indicates that daidzein and genistein were the main aglycones included in the samples. The highest content of daidzein was found in the yogurt alternatives obtained from soybeans (73.6 mg/100 g freeze-dried sample), while the lowest was found in those from white beans (1.2 mg/100 g freeze-dried sample). A similar trend was observed for genistein, which is consistent with previous studies showing significant amounts of isoflavone aglycones in soybeans and soy-based products compared to other vegetables [36]. The observed content of isoflavone aglycones could be influenced by both the raw material used and the production conditions of the yogurt alternatives. Moa et al. (2013) [37] reported differences in isoflavone content depending on the type of soybean; the yellow variety with black hilum contained significantly more genistein than the soybean with white hilum. In the production of bean-based yogurt alternatives, the seeds were sprouted, which could also affect the observed content of isoflavone aglycones. Guajardo-Flores et al. (2012) [38] showed that genistein was present in black bean seeds only after the third day of germination. The production conditions may have further influenced the observed isoflavone content. Previous research has shown that soaking enhances the leaching of these compounds into the water, fermentation reduces the content of isoflavone glucosides due to the activity of β-glucosidases produced by lactic acid bacteria, and cooking by boiling in water can cause a decrease of daidzein and genistein by up to 75% of the original value [36,37,38].

The anthocyanin content in the black bean-based yogurt alternatives was 0.22 mg/100 g, which is much lower than the values reported by Salinas-Moreno et al. (2005) [39] for 15 varieties of black beans grown in Mexico (37.7–71.6 mg/100 g). The main anthocyanins found in beans are delphinidin-3-*O*-glucoside (55% of total anthocyanins), followed by malvidin-3-*O*-glucoside (27%) and petunidin-3-*O*-glucoside (18%). This aligns with the findings of Takeoka et al. (1997) [40] who identified delphinidin-3-*O*-glucoside as the predominant anthocyanin in black beans but differs from the results reported by Mojica et al. (2017) [5] who found petunidin-3-*O*-glucoside to be the main anthocyanin in black bean extract. The difference between the current study’s results and the literature can be attributed to the conversion of all anthocyanins to malvidin-3-*O*-glucoside. The low content of anthocyanin in the plant-based yogurt alternatives, compared to black bean seeds, can be largely attributed to the significant addition of water into the product. In addition, the technological process of producing plant-based yogurt alternatives involves various operations that can significantly affect the anthocyanin content. The anthocyanin content in bean-based yogurt alternatives could also be affected by the processes of germination and fermentation [41].

Table 1 provides the content of phenolic acids in soy-based and bean-based yogurt alternatives. It shows that chlorogenic acid had the highest content in all the analyzed plant-based yogurt alternative samples. Most of the phenolic acids examined were detected in all samples, except for sinapic acid, which was not detected in soy-based yogurt alternatives. When comparing the content of individual phenolic acids, soy-based yogurt alternatives exhibited higher content of phenolic acids in each case, followed by black bean-based yogurt alternatives. The lowest content of phenolic acids was found in white bean-based yogurt alternatives, with an amount of 2.44 mg/100 g. Similarly, studies by Kim et al. (2006), [42] Xu and Chang (2008) [43], and Huber (2016) [44] reported that chlorogenic acid is the predominant phenolic acid found in soybeans and brown bean extracts. The content of phenolic acids in plant-based yogurt alternatives may be affected by the variety of soybeans and beans used in production, especially the color of their coat tissue. Kim et al. (2006) [42] showed that the concentration of phenolic compounds in brown and black soybeans was much higher compared to yellow or green soybeans. The fermentation process could also have influenced the content of phenolic acids in plant-based yogurts.

### 2.3. Changes in Anthocyanin Content during Storage of Strawberry and Blueberry Yogurts and Plant-Based Yogurt Alternatives

Changes in the content of anthocyanins during the storage of fermented products are presented in Table 2 and Table 3. Yogurts and plant-based yogurt alternatives with the addition of blueberry exhibited more than twice the amount of anthocyanins compared to those with the addition of strawberry. Blueberry yogurts had a total anthocyanin content of 24.46 mg/100 g, while strawberry yogurts had 9.48 mg/100 g. These results are comparable to those reported by Oliveira et al. (2015) [1] for yogurts with the addition of strawberry preparations. However, lower anthocyanin content was observed in the fermented blueberry products [4,16], which may be attributed to the addition of blueberry juice to yogurt rather than the blueberry preparation used in this study.

The main anthocyanin present in the products was pelargonidin-3-*O*-glucoside, accounting for 79% of the total anthocyanin content in strawberry yogurts (Figure S1A). Smaller amounts of pelargonidin-3-malonyl-glucoside, pelargonidin-3-*O*-rutinoside, and cyanidin-3-*O*-glucoside were also detected in yogurts and plant-based yogurt alternatives, representing 17%, 3%, and 2% of the total content of anthocyanin in strawberry yogurts, respectively. Previous studies [45,46] have documented that pelargonidin-3-*O*-glucoside is the dominant anthocyanin in strawberries and contributes the largest proportion to the total anthocyanin content. It has also been shown that strawberries contain 25 different anthocyanins and their content is influenced by factors such as variety, maturity, edaphic-climatic factors, and postharvest storage. The observed anthocyanin profile may also be affected by the production process of fruit preparation, especially the stages of fruit cooking, pasteurization, and thawing. Holzwarth et al. (2012) [47] observed that different thawing procedures significantly affected the anthocyanin content in strawberries, with the best retention achieved through thawing at 20 °C and in a microwave oven. Sadilova et al. (2006) [48] demonstrated that pelargonidin-3-*O*-glucoside isolated from strawberries was more stable than cyanidin-3-*O*-glucoside when heated at 95 °C and pH 1, although the differences were not significant.

An HPLC analysis of anthocyanins in blueberry yogurts and plant-based yogurt alternatives revealed the presence and quantification of 13 anthocyanins, which were glycosylated with three different monosaccharide moieties: galactose, glucose, and arabinose (Figure S1B). Additionally, acylated anthocyanins were detected but their specific identification was not provided. Table 3 presents changes in the total content of delphinidin, cyanidin, petunidin, and malvidin derivatives during the storage of fermented blueberry yogurts and plant-based yogurt alternatives. Peonidin derivatives are not included in the table due to their very low content. The effect of storage on the content of acylated anthocyanins in blueberry fermented products is also presented as the total sum. In general, malvidin derivatives and acylated anthocyanins were the most predominant forms in blueberry products, accounting for 55% and 16% of the overall anthocyanin content in blueberry yogurt, respectively. Delphinidin and petunidin derivatives were present at similar levels and accounted for approximately 12% of the total anthocyanin content. When comparing the obtained anthocyanin profile in blueberry plant-based yogurt alternative products with that reported by Giovanelli and Buratti (2009) [49] for fruits, differences can be observed. The fruit samples had a relatively higher content of delphinidin derivatives and a lower content of malvidin derivatives and acylated anthocyanins. Differences in the degree of ripeness of the examined fruits and the process of obtaining blueberry preparation could have had a significant impact on the observed anthocyanin profile. Chung et al. (2016) [2] showed that during the ripening of the Bluecrop variety, the process of anthocyanidin glycosylation occurs before methoxylation, resulting in the detection of only cyanidin and delphinidin in unripe blueberries. Furthermore, Sarkis et al. (2013) [50] showed that delphinidin derivatives are more unstable compared to malvidin derivatives during the heating of blueberry pulp, which may be related to the presence of hydroxyl substituents, which are more susceptible to degradation reactions.

The degradation of anthocyanins in strawberry and blueberry yogurts and plant-based yogurt alternatives during storage was analyzed using first-order reaction kinetics (Table 2 and Table 3). The rate of degradation was dependent on the concentration of anthocyanins, with a lower concentration resulting in a slower degradation rate [19]. The *k* values (rate constants) for total anthocyanin degradation ranged from 0.026 to 0.052/week for strawberry products and from 0.009 to 0.014/week for blueberry products. The half-life values, representing the time required for 50% degradation of total anthocyanins, ranged from 13.3 weeks in strawberry yogurts to 88.8 weeks in blueberry soy-based yogurt alternatives. The findings align with previous studies suggesting that anthocyanins in food products can follow first-order kinetics degradation. Baria et al. (2021) [11] reported *T*_1/2_ values of 285.6 days for anthocyanin degradation during the storage of black carrot yogurt alternatives, which is comparable to the results obtained for blueberry-fermented products. Similarly, Wallace and Giusti (2008) [19] found *T*_1/2_ values of 125 days during the storage of yogurts with the addition of 20 mg of *B. boliviana* extract, which aligns with the results observed for strawberry products in this study.

Significant differences in the stability of anthocyanins were observed depending on the fermented matrix of plant-based yogurt alternatives, both with the addition of strawberry and blueberry preparations. The highest *T*_1/2_ values for total anthocyanins were observed in fermented soybean matrix (26.3 and 88.8 weeks for strawberry and blueberry products, respectively), while the lowest values were found in yogurts (13.3 and 49.3 weeks for strawberry and blueberry products, respectively). When comparing the stability of anthocyanins during the storage of bean-based products, the colorants in the matrix obtained from black bean-based products showed better stability. These variations in anthocyanin stability may be attributed to multiple factors. Previous studies have demonstrated the significant effect of pH on the stability of anthocyanin, including the copigmentation effect of lactic acid through electrostatic bonding with colorants, and the protective effect of anthocyanins through water expulsion from the flavilium cation [8,9]. However, the examined products in this study had comparable pH and lactic acid levels (Table S3).

Differences in anthocyanin stability may also result from variations in sugar composition in the fermented matrices. Fruit yogurt samples contained higher amounts of glucose compared to plant-based yogurt alternatives (Table S3), and Kabakcı et al. (2020) [9] showed a negative correlation between glucose and anthocyanins during the storage of kefir with fruit juice, which is attributed to the effect of glucose on the precipitation of tannin compounds by protein. On the other hand, Wang et al. (2021) [51] reported glucose-protected anthocyanins in dried black goji fruit stored at low temperatures. Previous studies have also indicated that the presence of sugars in small amounts in food matrices can accelerate anthocyanin degradation, particularly leading to the formation of furfural and hydroxymethylfurfural during heat treatment. Fructose and lactose have a greater impact on anthocyanins degradation compared to glucose and sucrose [52].

The factor affecting the different stability of anthocyanins in the tested yogurt alternatives could be the different content and the composition of proteins contained in the fermented matrix, which can react with anthocyanins, form soluble complexes, or affect their stability during storage [1]. The highest protein content was found in yogurts and soy-based yogurt alternatives, in which the half-life of anthocyanins differed significantly. The formation of protein–polyphenolic complexes may be strongly influenced by the protein structure present in the matrix. Yogurts contain caseins and whey proteins, mainly β-lactoglobulin and α-lactalbumin, while soy-based yogurt alternatives contain globulins, mainly β-conglycinin, and glycinin [1,53]. Hsiao and Hsieh (2018) [54] showed interactions between β-conglycinin, glycinin, and anthocyanins during black soybean processing.

The different fat content in the fermented products could also contribute to the observed differences in anthocyanin stability. Soy-based yogurt alternatives had the highest fat content and exhibited the least degradation of anthocyanins during storage. On the other hand, yogurts had the same fat content as soy-based products but showed the greatest decrease in anthocyanins. Wallace and Giusti (2008) [19] reported that the stability of acylated anthocyanins in yogurts with the addition of purple carrots depended on the fat content, with significant differences in the half-life observed between yogurt with 4% fat content (550 days) and 0% fat content (129 days). However, such differences were not observed in the present study, which could be attributed to the use of different matrices.

The content of phenolic compounds in fruit plant-based yogurt alternatives differed from that of fruit yogurts (Table S3). This discrepancy could contribute to the observed differences in the stability of anthocyanins. The high content of isoflavone aglycones in fermented soy-based and black bean-based yogurt alternatives (Table 1) might have influenced the stability of anthocyanins, as suggested by a study conducted by Talcott et al. (2005) [55]. The study demonstrated that isoflavonoid extracts from red clover leaves improved the stability of 3,5-diglucosides present in grape juice and wine through intermolecular copigmentation reactions. Furthermore, soy-based and black bean-based yogurt alternatives contained significant amounts of ferulic and p-coumaric acids and research by Fan et al. (2019) [56] demonstrated that these acids act as effective copigments that enhance the stability of anthocyanins residues in blackberry-wine fermentation. Sotelo-González et al. (2023) [57] reported that polyphenols in the strawberry–blueberry blend beverage could exert a copigmentation effect, reducing the rate of anthocyanin degradation during storage.

When comparing the average half-life time (*T*_1/2_) of individual anthocyanins or anthocyanidin derivatives in all tested fermented products, it was found that the anthocyanins contained in the strawberry preparation exhibited similar stability, while significant differences were observed for the anthocyanins in the blueberry preparation. The average degradation time of anthocyanins in strawberry products ranged from 19 weeks for pelargonidin-3-*O*-glucoside to 23 weeks for pelargonidin-3-*O*-rutinoside. These results align with the research conducted by Ertan et al., (2018) [6] who showed that pelargonidin-3-*O*-rutinoside is characterized by higher stability than other anthocyanins during the storage of strawberry nectar. In blueberry products, the average half-life ranged from 36 weeks for delphinidin derivatives to 139 weeks for acylated anthocyanins. Much research has revealed that acylated anthocyanins show higher stability than their corresponding nonacylated anthocyanins. Acylation affects the stability of colorants in aqueous solutions through intramolecular copigmentation, proving protection to the flavylium chromophore against nucleophilic water attack and generating steric hindrance [58].

### 2.4. Changes in Color Parameters during Storage of Strawberry and Blueberry Yogurts and Plant-Based Yogurt Alternatives

Color is one of the most important quality criteria for fruit products. Changes in the values of color parameters are shown in Table 4 and Table 5. The tested products exhibited significant color differences. Yogurts with the addition of strawberries had the highest value of the L* parameter (69.4), indicating greater lightness, while the product obtained from black bean-based yogurt alternatives with blueberries had the lowest L* parameter value (30.9). The lightness of the products was largely influenced by the fermented matrix, particularly in products with blueberries added. Products obtained from milk were the lightest, while those derived from black beans appeared darker, with a brownish tint. The differences in lightness were also influenced by the type of fruit used in the preparation, with blueberry products being darker compared to their strawberry counterparts. Similar correlations were observed when comparing the lightness of the intermediate products, such as the fermented matrices (Table S2) and fruit preparations (Table S1). The highest color parameter a*, was characterized by the soy-based product, both with strawberry (14.4) and blueberry (13.8) preparations. Products obtained by bean fermentation were characterized by lower redness compared to others; the lowest a* parameter was recorded for the blueberry product obtained from white beans (9.1). The color parameter b* of the strawberry products immediately after production had positive values, while in the blueberry products, negative values. Strawberry yogurts had the highest degree of yellow coloration (b* = 6.0), while the fermented blueberry product obtained from soybeans had the lowest values (b* = −4.6), which had a bluish hue.

During the storage of yogurts and plant-based yogurt alternatives, it was observed that the L* color parameter significantly increased. However, no significant differences were observed in soy-based products with both strawberry and blueberry preparations, as well as in the white bean-based product with blueberry. In general, the storage time caused the products to lighten, a change that could be attributed to the degradation of anthocyanins. The a* color parameter gradually decreased during storage, resulting in a decrease in redness. The behavior of this parameter varied depending on the type of fruit filling. Strawberry products exhibited a significant decrease in the a* parameter during the second week of storage, while blueberry products showed a gradual decrease throughout the storage period. A similar decrease in the value of the color parameter a* was observed by Dias et al. (2020) [12] during the storage of soy-based yogurt alternatives with the addition of hibiscus. In contrast, the opposite relationship was observed when storing soy-based products with the addition of red radish, which suggests that the structure of anthocyanins is important.

Significant changes in the b* color parameter were also observed in some products during storage. In strawberry products, no significant changes were noted in the white bean-based product, while in other products, the b* parameter initially decreased until the 4th week of storage, and then increased to a higher value than in the products immediately after production by the 8th week of storage. In the case of products with a blueberry preparation, a gradual increase in color parameters was observed during storage, resulting in a shift towards less blueness and more yellowness. This could be attributed to the conversion of the colored flavylium cation of anthocyanins to colorless or yellowish forms.

In general, storing fermented products with fruit preparation caused a color change, particularly in strawberry products. The total color change after 8 weeks of storage in the plant-based yogurt alternatives with strawberry preparations exceeded a value of 2, making the color difference noticeable to the average observer [59]. The highest color difference values were recorded for strawberry yogurt, where the ΔE* after 8 weeks of storage reached 6.3. This indicates that the strawberry yogurt had distinctly different colors immediately after production compared to after 8 weeks of storage. For blueberry products, the observed differences were lower than those for strawberry products. However, the highest ΔE* values were still observed during the storage of blueberry yogurts. In the case of blueberry plant-based yogurt alternatives, the ΔE* was lower than two, indicating that differences in color between the product immediately after production and after 8 weeks of storage were noticeable only to an experienced observer [59].

Indeed, changes in the color of fermented fruit products during storage can also be attributed to changes in the white matrix. The white color of the milk is due to the reflection of light by dispersed fat globules, calcium caseinate, and calcium phosphate, as well as the presence of riboflavin (imparting a yellow color with green fluorescence to the whey of milk) and carotenoids including β-carotene, retinol, and xanthophylls (i.e., lutein and zeaxanthin) [60]. Samples may become lighter (higher L* values) due to the degradation of the natural milk colorants but also can be attributed to the different opacity levels of gel, which increased with the casein proportion and aggregation level [61]. A low storage temperature of yogurts can also inhibit nonenzymatic browning reactions, which would otherwise cause the darkening of the fermented products. In plant-based products, changes in the fermented matrix can also affect color. Beans contain flavonoids, including flavonol glycosides, anthocyanins, and proanthocyanidins (condensed tannins), which contribute to their color. These compounds can form complexes with proteins, metals, or polysaccharides, or undergo hydrolysis to form colored anthocyanins [62]. Studies conducted on soy beverages also show the influence of isoflavones content, especially glycitein on the color assessed by consumers [63].

## 3. Materials and Methods

### 3.1. Materials and Chemicals

The strawberries *Fragaria x ananassa* Duch. (Rumba variety) and highbush blueberries *Vaccinium corymbosum* L. (Bluecrop variety) used in the study were obtained from a local fruit grower near Warsaw (Poland). Upon harvest, the fruits were promptly frozen and stored at −35 °C until preparations were made. *Soybeans Glycine max* (L.) Merr., common beans *Phaseolus vulgaris* L. (white bean seeds—Piękny Jaś variety, black bean seeds—Midnight variety), sugar, and milk were purchased from a local market. Amidated apple pectin, citric acid, and modified corn starch were obtained from commercial producers. The culture of YC-X16 (containing *Streptococcus thermophilus* and *Lactobacillus delbrueckii* subsp. *bulgaricus*) was obtained from Chr. Hansen (Hoersholm, Denmark).

L-ascorbic acid, dithiothreitol, Folin–Ciocalteu’s reagent, phosphoric acid, lactose, glucose, fructose, saccharose, galactose, raffinose, stachyose, chlorogenic acid, p-coumaric acid, ferulic acid, daidzein, glycitein, and genistein were obtained from Sigma-Aldrich (Burlington, MA, USA). Malic acid, citric acid, and lactic acid were purchased from Merck KGAA (Darmstadt, Germany). Metaphosphoric, methanol, and acetonitrile were obtained from Honeywell Fluka (Charlotte, NC, USA). Cyanidin-3-*O*-arabinoside and malvidin-3-*O*-glucoside were purchased from LGC Standards (London, UK). Delphinidin-3-*O*-glucoside, cyanidin-3-*O*-glucoside, pelargonidin-3-*O*-glucoside, pelargonidin-3-*O*-rutinoside were obtained from Carbosynth Ltd. (Compton, UK), cyanidin-3-*O*-galactoside was purchased from Extrasynthese (Lyon, France). Malvidin-3-*O*-galactoside, delphinidin-3-*O*-galactoside, and petunidin-3-*O*-glucoside were obtained from Sigma-Aldrich (St. Louis, MO, USA). All solvents used were of analytical or HPLC grade. HPLC-grade water (18 MΩ) was prepared using a Millipore purification system.

### 3.2. Strawberry and Blueberry Preparations Production

The recipe for the fruit preparations was developed based on the previously determined soluble solids and total acidity of the strawberries and blueberries (Table S4). It was targeted to achieve a refractometric extract of 38°Brix, a total acidity of 0.65 g of citric acid/100 g of preparation, with the fruits constituting 60% of the preparation’s weight. Before formulating, the fruits were thawed at room temperature. The preparations were produced using a Buchi Rotavapor R-124 laboratory vacuum evaporator. The process involved adding fruits, half the weight of sugar, and water to the evaporator, followed by heating for 20 min under reduced pressure at 60 ± 2 °C. Subsequently, the pectin preparation and starch, which had been premixed with the remaining sugar and water according to the recipe, were added and then cooked for an additional 10 min. Toward the end of the cooking process, citric acid was incorporated. The soluble solids were adjusted to the desired value, and then the mixture was poured into jars and pasteurized at 85 °C for 10 min. During the heating process, two T-type thermocouples (MPI-CL-16-4, Metronic, Poland) were used to measure the temperature at the geometric center of the samples. The technological process of obtaining the fruit preparations was repeated three times, separately for each type of fruit.

### 3.3. Yogurts and Plant-Based Yogurts Alternatives Production

To produce the dairy yogurts, UHT milk with 2% fat (700 g) was carefully poured into sterile Schott bottles. The bottles were then immersed in a water bath and heated to a temperature of 45 °C. Once the milk reached the desired temperature, the starter culture YC-X16 (in sterile saline water) was added in an amount of 0.04%, following the recommendations of the manufacturer. The milk with the added starter culture was then transferred to an incubator and fermented at 45 °C for 5 h.

For the soy-based yogurt alternatives, a previously reported method was employed with a few modifications [64]. First, 500 g of soybeans were soaked in 1500 mL of deionized water at room temperature for about 10 h. The soaked soybeans were then washed several times with about 300 mL of water. The soaked and washed soybeans were homogenized using a CMP 250 V.V. blender (Robot Coupe, Vincennes, France) with 3 L of cold tap water. The resulting liquid was filtered through a large Buchner funnel, which was lined with three layers of cheesecloth and connected to a conical flask equipped with a vacuum pump (N 820.3, KNF Laboport, Freiburg-Munzingen, Germany). The filtered soy milk was adjusted to a final volume of 3000 mL using tap water. The soy milk was further homogenized using a two-stage homogenizer (TwinPanda 600, GEA, Parma, Italy) set at pressure settings of 80 bar for stage II and 200 bar for stage I. After homogenization, the soy milk was portioned into 700 g amounts in Schott bottles and sterilized by autoclaving at 121 °C for 15 min (2840EL Tuttnauer, New York, NY, USA). Once cooled, the reconstituted YC-X16 starter culture was added and the samples were incubated at 45 °C for 5 h.

The bean-based yogurt alternatives were prepared according to a method described by Ziarno et al. (2019) [65] with some adjustments. Whole and undamaged bean seeds were used, which were first germinated in a sprouter at 25 °C for 3 days, with daily moistening using tap water. After sprouting, the beans were placed in a Thermomix TM6 and ground with a small amount of tap water. Tap water was then added to achieve a seed-to-water ratio of 1:9, and the mixture homogenized. The homogenized liquid was strained through a 0.1 mm sieve and heated to 80 °C for 10 min to gelatinize the starch. Following boiling, 700 g of the liquid were poured into 1000 mL Schott bottles and sterilized in an autoclave at 121 °C for 20 min. The bean-based liquid, cooled to 45 °C, had the yogurt starter culture (reconstituted in sterile saline water) added. The mixture was thoroughly mixed and fermented in an incubator at 45 °C for 5 h.

To create the yogurts and plant-based yogurt alternatives with the inclusion of fruit preparations, strawberry and blueberry preparations were added at a ratio of 30% to the weight of the final product. The addition of fruit preparation had a visible effect on the texture of the mixed yogurt, slightly increasing its viscosity. This may be influenced by sugars, pectin substances, and starch contained in the fruit preparation, which improves the structure of the curd network in mixed yogurt. The mixtures were thoroughly combined and poured into sterilized glass jars (80 mL). The production process was replicated to yield 7 kg of each product type. The finished products were stored in a light-free environment at 4 ± 1 °C for 0, 2, 4, 6, and 8 weeks.

### 3.4. Physicochemical Analysis

The determination of soluble solids was conducted using a digital refractometer 30PX (Mettler Toledo, Columbus, OH, USA). The total titratable acidity of preparations was measured via potentiometric titration using a titrator TitroLine5000 (SI Analytics, Mainz, Germany) and a 0.1 N NaOH solution, according to the AOAC method, with slight modification for fruit products [66]. The sample was diluted with neutralized and boiled water, mixed with a magnetic stirrer, and then filtered using Whatman filter paper. The titration continued until a pH of 8.1 was achieved. The results were expressed as a percentage of citric acids, which is the predominant organic acid in the strawberry and blueberry preparations. The titratable acidity in the yogurt samples was determined according to the AOAC method [67] and the results were expressed in g of lactic acid/100 g of products. The pH value of the products was measured using a calibrated CPI-601 Elmetron pH electrode. The total nitrogen content of the samples was determined using the Kjeldahl method, as described in the AOAC [67]. The protein content was calculated using a conversion factor of 6.38 for milk yogurts, 5.71 for soy-based yogurts, and 6.25 for bean-based yogurt alternatives. The fat content was determined according to the ISO method [68]. Total phenolics were analyzed spectrophotometrically using the Folin–Ciocalteu reagent, following the method described by Gao et al. (2000) [69]. The extraction solvent used was a mixture of methanol, acetone, and water (350:350:300, *v*:*v*:*v*). In the case of yogurt, the pH of the samples was adjusted to pH 4.6 using 1 M HCL and the nonhydrolyzed casein was removed by centrifugation [70]. Absorption measurements were performed at 765 nm using a Shimadzu UV-1650PC spectrophotometer and the results were expressed as gallic acid equivalents. The color of the products was evaluated by measuring the L* (lightness), a* (redness/greenness), and b* (yellowness/blueness) parameters of the CIELab color system using a Konica Minolta 3600-d spectrophotometer. The instrument was calibrated against black and white plate standards. CIELab color space was obtained using a D65 illuminant and a 10° observation angle as the reference system, with a measured area diameter of 25.4 mm. The samples were placed in glass cuvettes with an optical length of 10 mm. Total color difference (ΔE*) represents the distance in three dimensions of the CIE Lab color space and was calculated using the following Equation (1)

(1) $$∆E^{*}=[[L_{t}^{*}-L_{0}^{*}{]}^{2}+{[a}_{t}^{*}-a_{0}^{*}{]}^{2}+{[b}_{t}^{*}-b_{0}^{*}{]}^{2}{]}^{1/2}$$

where, $L_{0}^{*},\;a_{0}^{*},\;b_{0}^{*}$ are the initial color parameters (t = 0) and $L_{t}^{*},\;a_{t}^{*},\;b_{t}^{*}$ are the values at time t [59].

### 3.5. Determination of Soluble Carbohydrates

The analysis of soluble carbohydrates in yogurt alternatives and fruit preparations followed a modified procedure based on the method described by Usenik et al. (2008) [71] and Elghali et al. (2005) [15]. Representative samples of the strawberry and blueberry preparations were homogenized and 5 g of each sample were extracted with 45 mL of distilled water for 30 min at 25 °C, with frequent stirring at 250 rpm (Unithermix, LLG Labware, Meckenheim, Germany). For the yogurt alternatives, 1 g of the samples was extracted with 9 mL of distilled water under the same conditions. After extraction, the samples were centrifuged for 10 min at 15,133× *g*. The resulting supernatants were filtered through a 0.45 µm nylon syringe filter and injected into an HPLC system. Chromatographic analyses were carried out using a Shimadzu Prominence HPLC system, which included an LC-20AD pump, autosampler (SIL-20A HT), refractive index detector (RID-10A), column oven (CTO-10ASVP), degasser (DGU-20A5R), and LABSolution software platform. A RezexTM RCM-Monosaccharide Ca+ column (300 × 7.8 mm) (Phenomenex, Torrance, CA, USA) was used for the separation of soluble carbohydrates. The column temperature was maintained at 75 °C and the mobile phase consisted of Milli-Q water with a flow rate of 0.6 mL/min. Sugars were quantified using the external standard method and the content was expressed as g/100 g of yogurt alternatives or fruit preparations.

### 3.6. Determination of Organic Acid

The determination of organic acid content followed a modified method based on the procedure described by Flores et al. (2012) [72]. Homogenized samples (2.5 g) were extracted with distilled water (10 mL) in a shaking thermostat. After 30 min of shaking at 25 °C, the extracts were centrifuged for 10 min at 15,133× *g* and 6 °C. This extraction process was repeated and the supernatants were combined to achieve a final volume of 25 mL with distilled water. The solutions were then passed through a 0.45 µm filter and a Sep-Pak^®^ C18 cartridge (previously washed with methanol and a phosphoric acid solution). The filtered samples (0.45 µm nylon syringe filter) were prepared for HPLC analysis. The analysis was performed using the previously mentioned Prominence chromatographic system, equipped with a Cosmosil 5C18-PAQ (4.6 mm × 150 mm) column and a diode array detector (SPD M20A). Elution was performed under isocratic conditions using a mobile phase of 20 mmol phosphoric acid at a flow rate of 1 mL/min and a temperature of 25 °C. A 20 µL injection volume was used and organic acid peaks were detected at either 210 or 254 nm. Quantification of organic acids was accomplished by comparing the peak area obtained with those of standard acids. The analysis was performed in triplicate and the content of organic acids was expressed as g/100 g of the products.

### 3.7. Determination of Ascorbic Acid and Dehydroascorbic Acid

The determination of vitamin C content was conducted using a modified method based on the procedure described by Chebrolu et al. (2012) [73] and Chotyakul et al. (2014) [74]. For yogurt samples, 5 g of yogurt were placed in a volumetric flask and diluted to 10 mL with a solution of 2.5% metaphosphoric acid. For the fruit products, 5 g of homogenized strawberry or 7 g of blueberry preparations were mixed with the extraction solvent (2.5% metaphosphoric acid) to achieve a final volume of 25 mL. The samples were transferred to the centrifuge tubes, vortexed for 2 min, and centrifuged at 15,133× *g* for 10 min. The resulting supernatants were filtered using a 0.45 µm syringe filter and then divided into two parts. One part of the supernatant was immediately analyzed for ascorbic acid content by mixing 300 µL of the filtered supernatant with 300 µL of 2.5% metaphosphoric acid and injecting it into the HPLC system. The other part of the supernatant (300 µL) was treated with dithiothreitol (300 µL, 10 mmol/L) and, after 30 min, the sample was injected into the HPLC system. The content of dehydroascorbic acid in the samples was calculated by the difference between the content of ascorbic acid (after the addition of dithiothreitol) and the initial content of ascorbic acid. Each sample was extracted in triplicate before HPLC analysis. The ascorbic acid analysis was analyzed using an HPLC system equipped with a UV-vis detector. Separation was achieved using an Onyx Monolithic column (100 × 4.6 mm) with a guard cartridge Onyx Monolithic C18 (10 × 4.6 mm, Phenomenex, Torrance, CA, USA). Elution was carried out under isocratic conditions with a flow rate of 1.0 mL/min at 25 °C using a 10 mM dihydrogen ammonium phosphate (pH 2.6) mobile phase. Ascorbic acid was detected at 254 nm and the results were expressed as mg/100 g of the product based on an ascorbic acid standard calibration curve (0–62 mg/L).

### 3.8. Determination of Isoflavone Aglycone

The content of isoflavone aglycones in the plant-based yogurt alternatives was determined using a modified liquid chromatography method, as described by da Costa César et al. (2006) [75]. Before analysis, the samples were freeze-dried using a laboratory freeze-dryer (Alpha 1-4 LSCplus, Christ, Osterode am Harz, Germany). A 50 mg portion of the freeze-dried sample was extracted with 45 mL of a 3.0 M HCl ethanolic solution. The solution was sonicated for 5 min and then heated (60 °C) for 45 min with frequent stirring at 250 rpm (Unithermix, LLG Labware, Meckenheim, Germany). After cooling, the sample was transferred to a 50 mL volumetric flask and brought to the mark. The samples were then centrifuged for 10 min at 15,133× *g* at 4 °C (MPW-350R, MPW Med. Instruments, Warsaw, Poland), and the supernatant was filtered using a 0.45 µm syringe filter before being injected into the HPLC systems. Chromatographic separation was carried out using the aforementioned HPLC system equipped with a diode array detector (SPD M20A) and a Luna C18(2) RP (5 μm) 250 × 4.6 column with a guard column (Phenomenex, Torrance, CA, USA). The mobile phase consisted of 0.1% acetic acid and methanol (520:480 *v:v*) and the flow rate was set at a constant 1 mL/min for 26 min at 30 °C. Detection was performed at 254 nm and the injection volume was 20 µL. The quantities of daidzein, glycitein, and genistein were determined using the corresponding calibration curves.

### 3.9. Determination of Phenolic Acids

The determination of phenolic acid content in the plant-based yogurt alternatives followed a modified HPLC method based on the procedure described by Oliveira et al. (2015) [1]. Samples of the plant-based yogurt (25 g) were homogenized with 25 mL of a methanol–formic acid solution (900:100 *v*/*v*) using an IKA Ultra-Turrax T18 Basic rotor-stator homogenizer. The solution was allowed to stand for 1 h at −24 °C to facilitate protein precipitation, followed by centrifugation (10 min at 15,133× *g* at 4 °C). The resulting samples were then filtered using a 0.45 µm PTFE membrane. The profiles of phenolic acids in the plant-based yogurt were determined using an HPLC-DAD method. Separation was achieved using a Luna C18(2) RP (5 μm) 250 × 4.6 column (Phenomenex, Torrance, CA, USA) fitted with a guard column (KJO-4282) at 25 °C. Gradient elution was employed with a mobile phase consisting of a formic acid–water solution 100:900 *v*/*v* (mobile phase A) and an acetonitrile–formic acid solution 900:100 *v*/*v* (mobile phase B) with the following gradient: 0–4 min, 8% B; 4–8 min, 8–20% B; 8–12 min, 20–65% B; 12–20 min, 65% B; 20–35 min, 65–8% B; 8% B for 8 min. The wavelengths used for the quantification of specific phenolic acid were 280 nm for *p*-coumaric acid and 320 nm for chlorogenic acid, ferulic acid, and sinapic acid. The injection volume was 20 µL and the flow rate of the mobile phase was 1 mL/min. The analyses were performed in triplicate and all phenolic acids were quantified using the external standard method.

### 3.10. Determination of Anthocyanins

To extract anthocyanins from the samples, 40 g of the sample were stirred with a mixture of 60 mL of methanol, water, and hydrochloric acid (700/300/1, *v*/*v*/*v*) for 10 min at 25 °C (Unithermix, LLG Labware, Meckenheim, Germany). The mixture was then sonicated at 25 °C and 37 kHz for an additional 10 min (ultrasonic bath, SW 3H, Sonoswiss AG, Ramsen, Switzerland). After centrifugation at 6880× *g* for 10 min, the residues were subjected to two additional extractions with 40 mL of the same solvent. The supernatants from each extraction were collected. The collected extracts were kept at −23 °C for 1 h to facilitate protein precipitation and then centrifuged at 15,133× *g* (4 °C) for an additional 5 min. The samples were evaporated in a rotary evaporator under a vacuum at 40 °C until the methanol was eliminated. The volume was adjusted to 25 mL using phosphoric acid (1.0 g/L). To remove sugars and organic acids, the extract was passed through a Sep-Pak^®^C_18_ cartridge that had been preconditioned with 10 mL of methanol followed by 10 mL of 0.1% phosphoric acid. A 10 mL portion of the extract was loaded onto the cartridge, washed with 10 mL of 0.1% phosphoric acid, and then eluted with 5 mL of methanol with 0.1% HCL. Samples were filtered through 0.45 μm PTFE filters before the analyses. An HPLC system equipped with a DAD detector was used for anthocyanin analysis. A Luna C18(2) RP (5 μm) 250 × 4.6 column with a guard column (Phenomenex, Torrance, CA, USA) was employed in the HPLC system, controlled by LABSolutions software version 5.97. Anthocyanins were eluted with a gradient of formic acid–water (mobile phase A, 100:900, *v*/*v*) and acetonitrile–formic acid (mobile phase B, 900:100, *v*/*v*) at a flow rate of 1 mL/min. The gradient elution for the strawberry products followed the pattern of 0–2 min at 9% B, 2–7 min at 9–20% B, 7–12 min at 20–50% B, 12–14 min at 50% B, 14–22 min at 50–9% B, and 9% B for 8 min. For the blueberry products, the gradient elution was as follows: 0–5 min at 5.5% B, 5–8 min at 5.5–9% B, 8–20 min at 9–11% B, 20–22 min at 11–14% B, 22–27 min at 14–22% B, 27–31 min at 22–35% B, 31–40 min at 35–5.5% B, and 5.5% B for 7 min. Anthocyanins were identified by comparing elution order and retention time to authentic standards or previously reported data. Pelargonidin-3-*O*-glucoside (for strawberry products) or malvidin-3-*O*-glucoside (for blueberry products) were used as external standards for quantification. The quantification of anthocyanins was performed at 520 nm and the content was expressed in mg/100 g of fermented product.

### 3.11. Degradation Kinetics of Anthocyanins as Affected by Storage Time

A linear relationship was obtained by plotting the log concentrations of total anthocyanins against the storage time (Figure S2). The values of the coefficient of determination ranged from 0.91 to 0.93 for strawberry products and from 0.83 to 0.87 for blueberry products. This kinetic type was expressed by the following Equations (2) and (3):

ln (C_t_/C_0_) = −k × t (2)

*T*_1/2_ = −ln 0.5 × k^−1^ (3)

where *C_t_* is anthocyanin concentration at time *t*, *C*_0_ is initial anthocyanin concentration (t = 0), *k* is the first-order reaction rate constant, and T_1/2_ is the half-life time [76].

### 3.12. Statistical Analyses

All the experiments were performed in triplicate and the results (Table 1, Table 2 and Table 3) were expressed as the mean ± standard deviation using the Statistica 13.3 software package (Tibco Inc., Palo Alto, CA, USA). To determine statistical differences among the kinetic parameters of anthocyanin degradation and the physicochemical composition of fruit preparations, yogurts, and plant-based yogurt alternatives, the data were analyzed by one-way analysis of variance followed by the Tukey HSD test at a significance level of *p* ≤ 0.05. Additionally, a two-way ANOVA was used to assess significant differences associated with the storage time and the type of fermented product.

## 4. Conclusions

In conclusion, this research confirms that the stability of the anthocyanin contained in the strawberry and blueberry preparations depends on the type of matrix used for fermentation. Soy-based and black bean-based matrices showed the least significant losses of anthocyanins, potentially due to their higher content of isoflavones. Additionally, it was observed that the stability of anthocyanins in the blueberry preparation was higher than in the strawberry preparation during cold storage. The half-life of the anthocyanins in blueberry products was significantly longer than in the strawberry products, which may be attributed to differences in the structure of the anthocyanins between these two fruits. Acylated anthocyanins and derivatives of malvidin and petunidin, which contain methoxy groups in the B-ring of the molecule, exhibited high stability. Considering the higher stability of strawberry anthocyanins contained in the soy-based yogurt alternatives, which are abundant in isoflavones, future studies could explore the interaction between anthocyanins and isoflavones to confirm their protective effect on pigments during refrigerated storage. Alongside the degradation of anthocyanins, changes in the color of stored yogurts and plant-based alternatives were noted. The products became lighter in color and the redness decreased, as indicated by an increase in the L* parameter and a decrease in the a* parameter. It should be noted that the color parameters immediately after production are influenced not only by the fruit preparation but also by the fermented matrix. Since the pH of the fermented matrices did not differ statistically, it is probably related to the presence of compounds such as anthocyanins in black beans or isoflavones and other polyphenols in soybeans and beans.

## Figures and Tables

**Table 1:** The content of isoflavone-aglycones, anthocyanins, and phenolic acid in plant-based yogurt alternatives *.

Isoflavone-Aglycones [mg/100 g of Freeze-Dried Sample]				
	Daidzein	Glycitein	Genistein	
Soy-based yogurt alternatives	73.6 ^a^ ± 4.4	18.2 ± 1.4	62.8 ^a^ ± 3.4	
White bean-based yogurt alternatives	1.2 ^c^ ± 0.2	nd	0.08 ^b^ ± 0.00	
Black bean-based yogurt alternatives	9.54 ^b^ ± 0.7	nd	0.67 ^b^ ± 0.03	
Anthocyanins [mg/100 g ^x^]				
	Delphinidin-3-*O*-glucoside	Petunidin-3-*O*-glucoside	Malvidin-3-*O*-glucoside	Total
Black bean-based yogurt alternatives	0.12 ± 0.01	0.04 ± 0.00	0.06 ± 0.01	0.22 ± 0.01
Phenolic acids [mg/100 g]				
	Chlorogenic acid	p-coumaric acid	Ferulic acid	Sinapic acid
Soy-based yogurt alternatives	4.12 ^a^ ± 0.3	3.40 ^a^ ± 0.6	3.37 ^a^ ± 0.6	nd
White bean-based yogurt alternatives	1.28 ^c^ ± 0.05	0.22 ^b^ ± 0.02	0.83 ^b^ ± 0.09	0.11 ^a^ ± 0.03
Black bean-based yogurt alternatives	1.74 ^b^ ± 0.09	0.39 ^b^ ± 0.05	1.05 ^b^ ± 0.05	0.14 ^a^ ± 0.07

* Data are expressed as mean ± standard deviation, ^x^ mg malvidin-3-glucoside equivalents/100 g of FW, nd—not detected, ^a–c^—means in the same column followed by different lowercase represent the significant difference (*p* ≤ 0.05).

**Table 2:** Changes in anthocyanin content during the storage of strawberry yogurts and plant-based yogurt alternatives and the kinetic parameters for the degradation of anthocyanins.

Anthocyanins	Product	Anthocyanin Content [mg/100 g]					Kinetic Parameters	
		Storage Time [Weeks]					−k [Week^−1^]	*T*_1/2_ [Week]
		0	2	4	6	8		
Cyanidin-3-*O*-glucoside	Yogurts	0.16 ^aA^	0.13 ^aB^	0.12 ^aBC^	0.11 ^aC^	0.11 ^aC^	0.044 ^v^	15.9 ^y^
	Soy-based yogurt alternatives	0.15 ^aA^	0.13 ^aB^	0.13 ^aB^	0.12 ^aB^	0.12 ^aB^	0.030 ^y^	23.3 ^v^
	White bean-based yogurt alternatives	0.15 ^aA^	0.13 ^aB^	0.12 ^aBC^	0.12 ^aBC^	0.11 ^aC^	0.038 ^x^	18.2 ^x^
	Black bean-based yogurt alternatives	0.15 ^aA^	0.13 ^aB^	0.12 ^aBC^	0.12 ^aBC^	0.11 ^aC^	0.039 ^x^	17.6 ^x^
Pelargonidin-3-*O*-glucoside	Yogurts	7.45 ^aA^	6.11 ^bB^	5.56 ^cC^	5.12 ^cD^	4.86 ^dE^	0.053 ^v^	13.0 ^z^
	Soy-based yogurt alternatives	7.08 ^cA^	6.37 ^aB^	6.12 ^aC^	5.87 ^aD^	5.76 ^aD^	0.026 ^z^	26.9 ^v^
	White bean-based yogurt alternatives	7.23 ^bA^	6.15 ^bB^	5.78 ^bC^	5.43 ^bD^	5.21 ^cE^	0.041 ^x^	16.9 ^y^
	Black bean-based yogurt alternatives	7.30 ^bA^	6.36 ^aB^	6.04 ^aC^	5.74 ^aD^	5.51 ^bE^	0.035 ^y^	19.7 ^x^
Pelargonidin-3-*O*-rutinoside	Yogurts	0.25 ^aA^	0.21 ^aB^	0.19 ^bBC^	0.18 ^bC^	0.18 ^cC^	0.041 ^v^	16.9 ^z^
	Soy-based yogurt alternatives	0.24 ^aA^	0.22 ^aB^	0.21 ^aBC^	0.20 ^aC^	0.20 ^aC^	0.023 ^x^	30.4 ^v^
	White bean-based yogurt alternatives	0.24 ^aA^	0.21 ^aB^	0.20 ^abBC^	0.19 ^abCD^	0.18 ^cD^	0.036 ^y^	19.3 ^x^
	Black bean-based yogurt alternatives	0.24 ^aA^	0.21 ^aB^	0.20 ^abBC^	0.20 ^aBC^	0.19 ^abC^	0.029 ^y^	23.7 ^y^
Pelargonidin *3*-malonyl-glucoside	Yogurts	1.62 ^aA^	1.36 ^aB^	1.25 ^aC^	1.16 ^cD^	1.11 ^cE^	0.047 ^v^	14.7 ^z^
	Soy-based yogurt alternatives	1.54 ^cA^	1.37 ^abB^	1.31 ^bC^	1.25 ^aD^	1.22 ^aD^	0.029 ^z^	23.8 ^v^
	White bean-based yogurt alternatives	1.60 ^abA^	1.38 ^bB^	1.30 ^bC^	1.22 ^bD^	1.17 ^bE^	0.039 ^x^	17.7 ^y^
	Black bean-based yogurt alternatives	1.57 ^bcA^	1.38 ^bB^	1.31 ^bC^	1.25 ^aD^	1.20 ^abE^	0.034 ^y^	20.6 ^x^
Total	Yogurts	9.48 ^aA^	7.81 ^bB^	7.13 ^cC^	6.58 ^dD^	6.25 ^dE^	0.052 ^v^	13.3 ^z^
	Soy-based yogurt alternatives	9.01 ^cA^	8.10 ^aB^	7.77 ^aC^	7.45 ^aD^	7.30 ^aD^	0.026 ^z^	26.3 ^v^
	White bean-based yogurt alternatives	9.23 ^bA^	7.87 ^bB^	7.40 ^bC^	6.95 ^cD^	6.68 ^cE^	0.040 ^x^	17.1 ^y^
	Black bean-based yogurt alternatives	9.27 ^bA^	8.08 ^aB^	7.68 ^aC^	7.30 ^bD^	7.01 ^bE^	0.034 ^y^	19.8 ^x^

^a–d^ Same column followed by different lowercase represents a significant difference (*p* ≤ 0.05). ^A–E^ Same line followed by different uppercase represents a significant difference (*p* ≤ 0.05). ^v,x,y,z^ Same column followed by different lowercase represents the significant difference (*p* ≤ 0.05). k-rate constant, *T*_1/2—_half-life time.

**Table 3:** Changes in anthocyanidin-derivatives content during storage of blueberry yogurts and plant-based yogurt alternatives and the kinetic parameters for the degradation.

Anthocyanidin Derivatives	Product	Anthocyanidin-Derivatives Content [mg/100 g]					Kinetic Parameters	
		Storage Time [Weeks]					−k [Week^−1^]	*T*_1/2_ [Week]
		0	2	4	6	8		
Delphinidin derivatives ^1^	Yogurts	3.00 ^aA^	2.70 ^bB^	2.59 ^cC^	2.51 ^cCD^	2.46 ^cD^	0.025 ^v^	27.9 ^y^
	Soy-based yogurt alternatives	2.94 ^aA^	2.74 ^bB^	2.65 ^bC^	2.60 ^bCD^	2.58 ^bD^	0.016 ^y^	42.5 ^v^
	White bean-based yogurt alternatives	3.00 ^aA^	2.73 ^bB^	2.63 ^b cC^	2.58 ^bCD^	2.55 ^bD^	0.020 ^x^	34.1 ^x^
	Black bean-based yogurt alternatives	3.10 ^aA^	2.85 ^aB^	2.77 ^aBC^	2.71 ^aCD^	2.68 ^aD^	0.018 ^y^	38.1 ^v^
Cyanidin derivatives ^2^	Yogurts	1.14 ^aA^	1.03 ^aB^	0.99 ^aB^	0.97 ^bBC^	0.96 ^bC^	0.021 ^v^	32.3 ^y^
	Soy-based yogurt alternatives	1.10 ^aA^	1.06 ^aAB^	1.03 ^aB^	1.01 ^abB^	1.00 ^abB^	0.012 ^y^	58.2 ^v^
	White bean-based yogurt alternatives	1.12 ^aA^	1.06 ^aAB^	1.01 ^aB^	1.00 ^abB^	1.00 ^abB^	0.014 ^x^	48.9 ^x^
	Black bean-based yogurt alternatives	1.14 ^aA^	1.08 ^aB^	1.05 ^aBC^	1.04 ^aBC^	1.03 ^aC^	0.013 ^y^	54.6 ^v^
Petunidin derivatives ^3^	Yogurts	2.90 ^abA^	2.76 ^bB^	2.68 ^bBC^	2.65 ^bC^	2.64 ^bC^	0.012 ^x^	59.0 ^x^
	Soy-based yogurt alternatives	2.85 ^bA^	2.76 ^bAB^	2.71 ^abB^	2.67 ^abB^	2.66 ^bB^	0.009 ^y^	80.4 ^v^
	White bean-based yogurt alternatives	2.88 ^bA^	2.70 ^bB^	2.59 ^cC^	2.54 ^cC^	2.53 ^cC^	0.016 ^v^	42.8 ^y^
	Black bean-based yogurt alternatives	3.00 ^aA^	2.85 ^aB^	2.79 ^aBC^	2.77 ^aBC^	2.75 ^aC^	0.011 ^x y^	63.7 ^x^
Malvidin derivatives ^4^	Yogurts	13.56 ^aA^	12.75 ^cB^	12.50 ^bC^	12.25 ^cD^	12.21 ^cD^	0.013 ^v^	52.9 ^z^
	Soy-based yogurt alternatives	13.43 ^bA^	13.03 ^aB^	12.90 ^aC^	12.77 ^aD^	12.75 ^aD^	0.006 ^y^	106.7 ^v^
	White bean-based yogurt alternatives	13.43 ^bA^	12.89 ^bB^	12.50 ^bC^	12.38 ^bD^	12.34 ^bD^	0.011 ^x^	65.5 ^y^
	Black bean-based yogurt alternatives	13.50 ^abA^	12.96 ^abB^	12.83 ^aC^	12.71 ^aD^	12.69 ^aD^	0.007 ^y^	89.6 ^x^
Acylated anthocyanins ^5^	Yogurts	3.83 ^aA^	3.67 ^bB^	3.64 ^bBC^	3.60 ^cBC^	3.59 ^cC^	0.008 ^v^	85.7 ^x^
	Soy-based yogurt alternatives	3.79 ^aA^	3.71 ^abAB^	3.68 ^abB^	3.66 ^bB^	3.65 ^bB^	0.005 ^y^	147.3 ^x^
	White bean-based yogurt alternatives	3.83 ^aA^	3.74 ^abB^	3.70 ^abB^	3.67 ^bB^	3.65 ^bB^	0.006 ^x^	115.2 ^y^
	Black bean-based yogurt alternatives	3.83 ^aA^	3.79 ^aAB^	3.75 ^aBC^	3.74 ^aC^	3.73 ^aC^	0.003 ^z^	209.6 ^v^
Total	Yogurts	24.46 ^aA^	22.92 ^cB^	22.39 ^cC^	21.98 ^dD^	21.86 ^dE^	0.014 ^v^	49.3 ^z^
	Soy-based yogurt alternatives	24.11 ^bA^	23.29 ^bB^	22.96 ^bC^	22.70 ^bD^	22.65 ^bD^	0.008 ^y^	88.8 ^y^
	White bean-based yogurt alternatives	24.26 ^abA^	23.12 ^bcB^	22.45 ^cC^	22.17 ^cD^	22.07 ^cD^	0.012 ^x^	58.6 ^x^
	Black bean-based yogurt alternatives	24.56 ^aA^	23.53 ^aB^	23.18 ^aC^	22.96 ^aD^	22.88 ^aD^	0.009 ^y^	78.3 ^y^

^a–d^ Same column followed by different lowercase represents a significant difference (*p* ≤ 0.05). ^A–E^ Same line followed by different uppercase represents a significant difference (*p* ≤ 0.05). ^v,x,y,z^ Same column followed by different lowercase represents the significant difference (*p* ≤ 0.05). k-rate constant, *T*_1/2—_half-life time. ^1^ Delphinidin derivatives: delphinidin-3-*O*-galactoside, delphinidin-3-*O*-glucoside, delphinidin-3-*O*-arabinoside. ^2^ Cyanidin derivatives: cyanidin-3-*O*-galactoside, cyanidin-3-*O*-glucoside, cyanidin-3-*O*-arabinose. ^3^ Petunidin derivatives: petunidin-3-*O*-galactoside, petunidin-3-*O*-glucoside, petunidin-3-*O*-arabinoside. ^4^ Malvidin derivatives: malvidin-3-*O*-galactoside, malvidin-3-*O*-glucoside, malvidin-3-*O*-arabinoside. ^5^ Acylated anthocyanins.

**Table 4:** Evolution of CIELab color parameters and total color difference (ΔE*) of yogurts and plant-based yogurt alternatives with strawberry preparations during storage.

Color Parameters	Product	Storage Time [Weeks]				
		0	2	4	6	8
L*	Yogurts	69.4 ^aD^	70.2 ^aCD^	71.4 ^aC^	73.4 ^aB^	74.1 ^aA^
	Soy-based yogurt alternatives	64.5 ^bA^	64.4 ^bA^	64.3 ^bA^	64.2 ^bA^	64.5 ^bA^
	White bean-based yogurt alternatives	57.8 ^cB^	57.9 ^cB^	58.3 ^cAB^	58.3 ^cAB^	58.6 ^cA^
	Black bean-based yogurt alternatives	38.4 ^dB^	39.5 ^dAB^	39.6 ^dAB^	40.3 ^dA^	40.6 ^dA^
a*	Yogurts	13.6 ^bA^	10.5 ^bB^	10.4 ^bB^	10.3 ^bB^	9.8 ^bC^
	Soy-based yogurt alternatives	14.4 ^aA^	12.6 ^aB^	12.6 ^aB^	12.6 ^aB^	12.1 ^aC^
	White bean-based yogurt alternatives	11.9 ^cA^	10.1 ^bB^	9.5 ^cC^	9.5 ^cC^	9.4 ^bC^
	Black bean-based yogurt alternatives	10.3 ^dA^	9.9 ^cAB^	9.7 ^cB^	9.3 ^cC^	8.5 ^cD^
b*	Yogurts	6.0 ^aBC^	5.7 ^aC^	4.8 ^aD^	6.3 ^aB^	7.6 ^aA^
	Soy-based yogurt alternatives	4.7 ^bA^	4.6 ^bA^	4.2 ^bB^	4.8 ^bA^	4.9 ^bA^
	White bean-based yogurt alternatives	4.2 ^cA^	4.2 ^bA^	4.2 ^bA^	4.3 ^cA^	4.4 ^cA^
	Black bean-based yogurt alternatives	4.5 ^bcAB^	3.6 ^cC^	3.1 ^cD^	4.2 ^cB^	4.7 ^bcA^
ΔE*	Yogurts	-	3.2 ^aD^	4.0 ^aC^	5.2 ^aB^	6.3 ^aA^
	Soy-based yogurt alternatives	-	1.8 ^bB^	1.9 ^cAB^	1.8 ^cB^	2.2 ^cA^
	White bean-based yogurt alternatives	-	1.8 ^bB^	2.5 ^bA^	2.5 ^bA^	2.6 ^bA^
	Black bean-based yogurt alternatives	-	1.5 ^cC^	1.9 ^cB^	2.2 ^bB^	2.8 ^bA^

^a–d^ Same column followed by different lowercase represents the significant difference (*p* ≤ 0.05). ^A–D^ Same line followed by different uppercase represents the significant difference (*p* ≤ 0.05).

**Table 5:** Evolution of CIELab color parameters and total color difference (ΔE*) of yogurts and plant-based yogurt alternatives with blueberry preparations during storage.

Color Parameters	Product	Storage Time [Weeks]				
		0	2	4	6	8
L*	Yogurts	56.2 ^aC^	56.3 ^aC^	56.8 ^aB^	57.6 ^aA^	57.8 ^aA^
	Soy-based yogurt alternatives	46.8 ^bA^	46.7 ^bA^	46.8 ^bA^	46.9 ^bA^	46.9 ^bA^
	White bean-based yogurt alternatives	39.3 ^cA^	39.3 ^cA^	39.4 ^cA^	39.4 ^cA^	39.5 ^cA^
	Black bean-based yogurt alternatives	30.9^dC^	31.1^dC^	31.7 ^dB^	31.8 ^dAB^	32.1 ^dA^
a*	Yogurts	12.8 ^bA^	12.4 ^bAB^	12.1 ^bB^	11.6 ^bB^	11.4 ^bB^
	Soy-based yogurt alternatives	13.8 ^aA^	13.6 ^aAB^	13.3 ^aBC^	12.9 ^aCD^	12.7 ^aD^
	White bean-based yogurt alternatives	9.1 ^cA^	8.7 ^dB^	8.6 ^dBC^	8.4 ^dCD^	8.2 ^dD^
	Black bean-based yogurt alternatives	9.4 ^cA^	9.1 ^cAB^	9.2 ^cAB^	8.7 ^cB^	8.7 ^cB^
b*	Yogurts	−3.6 ^cC^	−3.4 ^cBC^	−3.0 ^cAB^	−2.9 ^cA^	−2.8 ^cA^
	Soy-based yogurt alternatives	−4.6 ^dB^	−4.5 ^dB^	−4.1 ^dAB^	−4.0 ^dA^	−3.9 ^dA^
	White bean-based yogurt alternatives	−2.6 ^bC^	−2.5 ^bBC^	−2.5 ^bBC^	−2.2 ^bAB^	−2.0 ^bA^
	Black bean-based yogurt alternatives	−0.7 ^aB^	−0.7 ^aB^	−0.4 ^aAB^	−0.2 ^aA^	−0.2 ^aA^
ΔE*	Yogurts	−	0.5 ^aC^	1.1 ^aB^	2.0 ^aA^	2.3 ^aA^
	Soy-based yogurt alternatives	−	0.2 ^bC^	0.7 ^bcB^	1.1 ^bAB^	1.3 ^bcA^
	White bean-based yogurt alternatives	−	0.4 ^abC^	0.5 ^cBC^	0.8 ^cAB^	1.1 ^cA^
	Black bean-based yogurt alternatives	−	0.4 ^abC^	0.9 ^abB^	1.2 ^bAB^	1.5 ^bA^

^a–d^ Same column followed by different lowercase represents the significant difference (*p* ≤ 0.05). ^A–D^ Same line followed by different uppercase represents the significant difference (*p* ≤ 0.05).

## Data Availability

All data analyzed during this study are included in this published article.

## Supplementary Materials

The following supporting information can be downloaded at: https://www.mdpi.com/article/10.3390/molecules28176222/s1, Figure S1. HPLC chromatogram of anthocyanin profile of strawberry (A) and blueberry (B) preparations. Figure S2. First-order degradation plots of total anthocyanins for strawberry (A) and blueberry (B) products storage during 8 weeks. Table S1. Physicochemical composition of strawberry and blueberry preparations *. Table S2. Physicochemical properties of yogurts and plant-based yogurt alternatives *. Table S3. Physicochemical properties of fruit yogurts and plant-based yogurt alternatives directly after production *. Table S4. The recipe for fruit preparations.
