# Supplementary material for: Effect of Fermented Matrix on the Color and Stability of Strawberry and Blueberry Anthocyanins during the Storage of Fruit Yogurts and Soy-Based and Bean-Based Fruit Yogurt Alternatives
Source: Molecules. 2023 Aug 24;28(17):6222. doi: 10.3390/molecules28176222 (PMC10488363; doi:10.3390/molecules28176222)

Table S1. Physicochemical composition of strawberry and blueberry preparations*.

Parameters		Strawberry preparation	Blueberry preparation
Soluble solids [°Bix]		$38.4^{a} \pm 0.5$	$38.9^{a} \pm 0.8$
Titratable acidy [g/100 g]		$0.68^{a} \pm 0.08$	$0.70^{a} \pm 0.05$
pH		$3.52^{b} \pm 0.01$	$3.62^{a} \pm 0.02$
Colorimetric parameters	L*	$29.4^{a} \pm 0.9$	$24.7^{b} \pm 1.2$
	a*	$8.1^{b} \pm 0.6$	$10.9^{a} \pm 0.7$
	b*	$2.2^{b} \pm 0.3$	$0.4^{a} \pm 0.3$
Fructose [g/100 g]		$8.4^{b} \pm 0.5$	$12.0^{a} \pm 1.1$
Glucose [g/100 g]		$8.1^{b} \pm 0.8$	$11.5^{a} \pm 0.9$
Sucrose [g/100 g]		$20.7^{a} \pm 1.5$	$13.8^{b} \pm 0.8$
Citric acid [g/100 g]		$0.51^{b} \pm 0.08$	$0.68^{a} \pm 0.07$
Malic acid [g/100 g]		$0.143^{a} \pm 0.02$	$0.012^{b} \pm 0.002$
L-ascorbic acid [mg/100 g]		$19.5^{a} \pm 1.2$	$1.5^{b} \pm 0.2$
L-dehydroascorbic acid [mg/100 g]		$3.1^{a} \pm 0.1$	$1.2^{b} \pm 0.04$
Phenolic content [mg/100 g]		$116.4^{b} \pm 2.8$	$157.0^{a} \pm 3.3$

* Data are expressed as mean ± standard deviation, $^{a-b}$ - means in the same row followed by different lowercase represent the significant difference ($p \le 0.05$)

Table S2. Physicochemical properties of yogurts and plant-based yogurt alternatives*.

Parameters		Yogurts	Soy-based yogurt alternatives	White bean-based yogurt alternatives	Black bean-based yogurt alternatives
pH		$4.41^{a} \pm 0.6$	$4.55^{a} \pm 0.7$	$4.57^{a} \pm 0.5$	$4.46^{a} \pm 0.8$
Colorimetric parameters	L*	$90.3^{a} \pm 1.8$	$83.6^{b} \pm 2.4$	$61.3^{c} \pm 1.9$	$43.4^{d} \pm 1.0$
	a*	$0.7^{c} \pm 0.1$	$-0.12^{d} \pm 0.02$	$2.9^{b} \pm 0.3$	$7.4^{a} \pm 0.5$
	b*	$10.9^{b} \pm 0.7$	$11.6^{b} \pm 1.2$	$16.8^{a} \pm 1.4$	$4.9^{c} \pm 0.5$
Glucose [g/100 g]		$0.31^{a} \pm 0.02$	$0.03^{b} \pm 0.004$	$0.02^{b} \pm 0.003$	$0.02^{b} \pm 0.001$
Sucrose [g/100 g]		nd	$0.27^{a} \pm 0.01$	$0.13^{b} \pm 0.01$	$0.09^{c} \pm 0.004$
Lactose [g/100 g]		2.76 ± 0.16	nd	nd	nd
Galactose [g/100 g]		$1.05^{a} \pm 0.12$	$0.21^{b} \pm 0.04$	$0.18^{b} \pm 0.03$	$0.15^{b} \pm 0.06$

Raffinose [g/100 g]	nd	0.09^{a} ± 0.007	0.04^{b} ± 0.008	0.03^{b} ± 0.002
Stachyose [g/100 g]	nd	0.34^{a} ± 0.01	0.18^{b} ± 0.02	0.12^{c} ± 0.01
Lactic acid [g/100 g]	0.60^{a} ± 0.01	0.55^{b} ± 0.02	0.53^{b} ± 0.01	0.55^{b} ± 0.01
L-ascorbic acid [mg/100 g]	0.38^{c} ± 0.01	0.25^{d} ± 0.02	0.41^{b} ± 0.02	1.5^{a} ± 0.00
L-dehydroascorbic acid [mg/100 g]	0.02^{b} ± 0.001	0.01^{c} ± 0.00	0.03^{a} ± 0.001	0.03^{a} ± 0.002
Protein content [%]	3.3^{a} ± 0.4	3.2^{a} ± 0.6	2.0^{b} ± 0.3	2.2^{b} ± 0.1
Fat content [%]	2.1^{a} ± 0.3	2.1^{a} ± 0.2	0.15^{b} ± 0.04	0.13^{b} ± 0.03

* Data are expressed as mean ± standard deviation. nd – not detected, $^{a-d}$ - means in the same row followed by different lowercase represent the significant difference (p≤0.05)

Table S3. Physicochemical properties of fruit yogurts and plant-based yogurt alternatives directly after production*.

Parameters	Fruit preparation	Yogurts	Soy-based yogurt alternatives	White bean-based yogurt alternatives	Black bean-based yogurt alternatives
pH	Strawberry	4.15^{a} ± 0.2	4.23^{a} ± 0.3	4.25^{a} ± 0.3	4.19^{a} ± 0.3
	Blueberry	4.17^{a} ± 0.2	4.25^{a} ± 0.3	4.26^{a} ± 0.2	4.19^{a} ± 0.2
Titratable acidy [g/100 g]	Strawberry	0.64^{a} ± 0.04	0.59^{a} ± 0.02	0.60^{a} ± 0.02	0.61^{a}± 0.02
	Blueberry	0.66^{a} ± 0.06	0.60^{a} ± 0.01	0.58^{a} ± 0.02	0.63^{a}± 0.05
Fructose [g/100 g]	Strawberry	2.49^{a} ± 0.2	2.46^{a} ± 0.5	2.48^{a} ± 0.3	2.46^{a} ± 0.1
	Blueberry	3.55^{a} ± 0.1	3.59^{a} ± 0.2	3.58^{a} ± 0.0	3.60^{a} ± 0.2
Glucose [g/100 g]	Strawberry	2.89^{a} ± 0.1	2.41^{b} ± 0.0	2.40^{b} ± 0.2	2.39^{b} ± 0.0
	Blueberry	3.59^{a} ± 0.1	3.40^{b} ± 0.0	3.41^{b} ± 0.1	3.39^{b} ± 0.2
Sucrose [g/100 g]	Strawberry	6.18^{c} ± 0.03	6.37^{a} ± 0.00	6.29^{b} ± 0.03	6.27^{b} ± 0.03
	Blueberry	4.15^{b} ± 0.04	4.38^{a} ± 0.08	4.20^{b} ± 0.02	4.19^{b} ± 0.06
Lactose [g/100 g]	Strawberry	1.91 ± 0.04	nd	nd	nd
	Blueberry	1.90 ± 0.08	nd	nd	nd
Galactose [g/100 g]	Strawberry	0.73^{a}± 0.09	0.14^{b}± 0.08	0.11^{b}± 0.03	0.11^{b}± 0.05
	Blueberry	0.72^{a}± 0.10	0.16^{b}± 0.04	0.13^{b}± 0.07	0.99^{b}± 0.04
Raffinose	Strawberry	nd	0.061^{a} ± 0.005	0.026^{b} ± 0.003	0.020^{b} ± 0.001

[g/100 g]	Blueberry	nd	0.063^{a} ± 0.004	0.026^{b} ± 0.005	0.019^{b} ± 0.006
Stachyose [g/100 g]	Strawberry	nd	0.23^{a}± 0.02	0.12^{b}± 0.01	0.08^{c}± 0.01
	Blueberry	nd	0.24^{a}± 0.01	0.12^{b}± 0.00	0.07^{c}± 0.01
Citric acid [g/100 g]	Strawberry	0.15^{a}± 0.04	0.14^{a}± 0.05	0.14^{a}± 0.03	0.16^{a}± 0.08
	Blueberry	0.21^{a}± 0.06	0.19^{a}± 0.05	0.19^{a}± 0.05	0.19^{a}± 0.08
Lactic acid [g/100 g]	Strawberry	0.42^{a} ± 0.00	0.37^{b} ± 0.01	0.36^{b} ± 0.02	0.36^{b} ± 0.00
	Blueberry	0.43^{a} ± 0.01	0.36^{b} ± 0.01	0.35^{b} ± 0.02	0.34^{b} ± 0.02
Malic acid [g/100 g]	Strawberry	0.04^{a} ± 0.02	0.04^{a} ± 0.02	0.03^{a} ± 0.00	0.04^{a} ± 0.01
	Blueberry	0.004^{a} ± 0.00	0.003^{a} ± 0.01	0.004^{a} ± 0.00	0.004^{a} ± 0.00
Phenolic content [mg/100 g]	Strawberry	35.9^{c} ± 1.5	55.5^{a} ± 1.0	42.1^{b} ± 1.2	54.5^{a} ± 1.1
	Blueberry	48.2^{c} ± 0.9	66.1^{a} ± 1.3	51.4^{b} ± 0.8	62.8^{a} ± 1.4

* Data are expressed as mean ± standard deviation. nd – not detected, $^{a-d}$ - means in the same row followed by different lowercase represent the significant difference (p≤0.05).

Table S4. The recipe for fruit preparations.

Product	Fruit preparation [1000 g]					
	Fruit [g]	Sugar [g]	Citric acid [g]	Water [g]	Pectin preparation [g]	Starch [g]
Strawberry preparation	600	324	3	66	5	2
Blueberry preparation	600	301	3.5	88.5	5	2

Figure 1S. HPLC chromatogram of anthocyanin profile of strawberry (A) and blueberry (B) preparations.

A

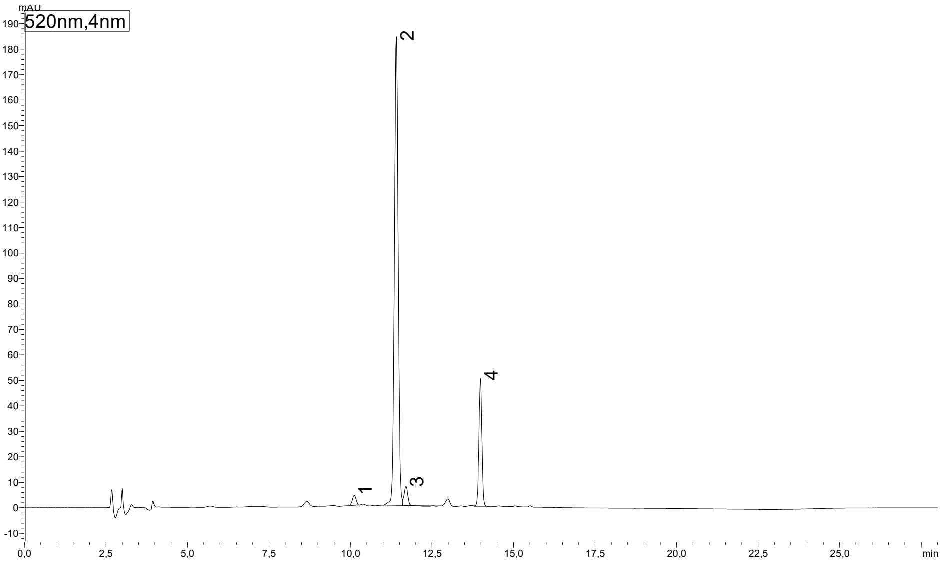

1.Cyanidin-3-*O*-glucoside, 2.Pelargonidin-3-*O*-glucoside, 3.Pelargonidin-3-*O*-rutinoside, 4. Pelargonidin *3*-malonyl-glucoside.

B

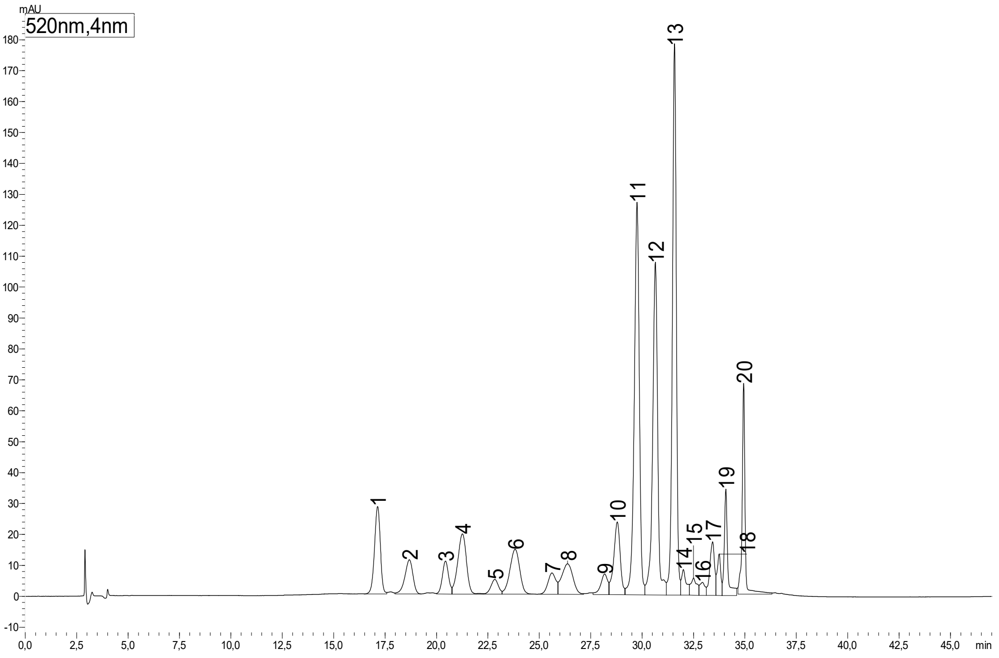

1.Delphinidin-3-*O*-galactoside, 2. Delphinidin-3-*O*-glucoside, 3. Cyanidin-3-*O*-galactoside, 4. Delphinidin-3-*O*-arabinoside, 5. Cyanidin-3-*O*-glucoside, 6. Petunidin-3-*O*-galactoside, 7. Cyanidin-3-*O*-arabinoside, 8. Petunidin-3-*O*-glucoside, 9. Peonidin-3-*O*-galactoside, 10. Petunidin-3-*O*- arabinoside, 11. Malvidin-3-*O*-galactoside, 12. Malvidin-3-*O*-glucoside, 13. Malvidin-3-*O*-arabinoside, 14-20 - Acylated anthocyanins.

Figure 2S. First-order degradation plots of total anthocyanins for strawberry (A) and blueberry (B) products storage during 8 weeks.

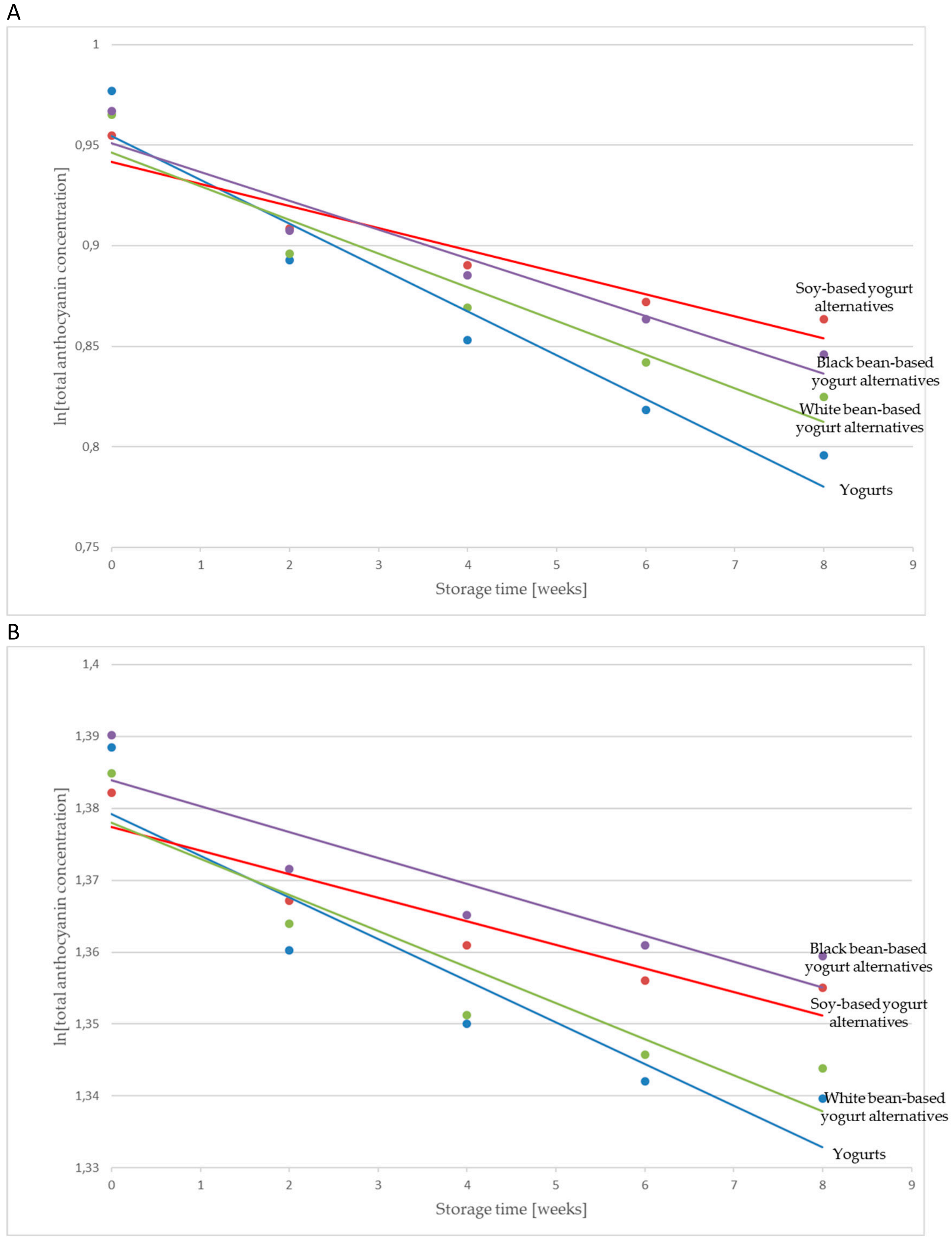

Supplement: Supplementary file 1 [file molecules-28-06222-s001.zip › molecules-2544646-supplementary.pdf]
